# Supplementary material for: Are carbon emissions trading and green financial instruments synergistic? -Comprehensive quantitative research based on content analysis
Source: PLoS One. 2024 Mar 7;19(3):e0298601. doi: 10.1371/journal.pone.0298601 (PMC10919601; doi:10.1371/journal.pone.0298601)
Supplement: S1 Data — (ZIP) [file pone.0298601.s001.zip › Data/地级市Z_F工作报告.zip!/╔╜╬≈/╦╖╓▌╩╨/└┤╫╘╣1⁄2╓┌║┼í╛┬φ┐╦╩2╛▌═°í┐_2009.docx]

各位代表:

　　现在，我代表市人民政府向大会报告本届政府过去五年的工作，对下届政府工作和2009年总体部署提出建议，请予审议，并请市政协各位委员和其他列席人员提出意见。

　　一、过去五年工作回顾

　　过去五年，在省委、省政府和市委的正确领导及市人大、市政协的有力监督支持下，市人民政府坚持以邓小平理论和"三个代表"重要思想为指导，认真贯彻落实科学发展观，团结带领全市人民，大力弘扬"豪爽大气、海纳百川、百折不挠、奋力赶超"的朔州精神，解放思想，奋发进取，全市经济社会实现了超常规跨越式大发展，成为建市以来经济社会发展最快、效益最好、人民群众得到实惠最多的时期。

　　国民经济持续快速发展，综合经济实力跨上新台阶。2008年，全市地区生产总值达到420.4亿元，年均增长14.7始，绝对额从全省第十一位前移到第九位。财政总收入达到102亿元，是2003年的6.9倍，年均增长47.3%，绝对额从全省第十位前移到第八位;一般预算收入达到37.8亿元，是2003年的7.16倍，年均增长48.2%。工业增加值达到242.6亿元，年均增长20.8%。固定资产投资五年累计607.8亿元，年均增长34.4%。城镇居民人均可支配收入达到13997元，高出全省平均水平878元，比2003年翻了近一番;农民人均纯收入达到4732元，高出全省平均水平635元，净增2200多元;社会消费品零售总额达到104.4亿元，年均增长21.6%。

　　招商引资成效显著，产业结构调整实现重大突破。坚持以大开放促进大发展，招商引资工作一直走在全省前列，近三年就有284个项目落地，引进资金271亿元。产业结构调整步伐越来越快。工业结构进一步优化。煤炭工业集约化、规模化、机械化水平明显提高，可持续发展能力大为增强。地方煤矿由207座压减到135座，单井生产规模由15万吨提高到45万吨，原煤生产能力由3040万吨提高到1.25亿吨，全省第一;22座煤矿实现了机械化综采，煤炭回采率由20%提高到70%左右;煤炭洗选能力由6000万吨提高到1亿多吨。电力工业快速发展。大唐、平朔、永浩、格瑞特四家电厂建成投产;平鲁、右玉两座风力电场投入运营，填补了全省空白。电力装机容量由241.8万千瓦增加到455万千瓦，位居全省前列。以煤电为基础，煤电冶、煤建材、煤化工等循环经济产业链正在形成。新兴产业加速成长。日用瓷生产能力由7亿件提高到10.5亿件，成为全国最大的日用瓷生产基地。乳品加工能力由42万吨提高到80万吨。冶金、化工、制药、新型材料等产业得到发展壮大。现代服务业快速起步。2008年，我们举全市之力，大打服务业攻坚战，当年开工建设了160个服务业项目，完成投资73亿元，旅游业开发大规模展开，现代物流业迅速扩张，大型机电设备维修业正在形成规模，

　　服务业增加值占地区生产总值的比重一年提高1.5个百分点。

　　农业综合生产能力全面提高，新农村建设稳步推进。不断加大对三农"的支持力度，在不折不扣执行上级各项强农惠农政策的基础上，市财政累计投入22亿元，对小杂粮、甜菜、大棚种植、养殖小区建设等连续实行补贴。积极加快现代农业发展，扎实推进玉米、马铃薯、小杂粮、葫麻、甜菜、蔬菜六大种植基地建设，大力引进现代农业科学技术，加强农业基础设施和服务体系建设，农业生产水平不断提高，粮食生产连续五年刷新历史纪录。进一步加强生态畜牧基地建设，实施了"亿元惠民工程"，全市奶牛存栏由9.5万头增加到14.4万头，占据全省半壁江山，并建成全国重要肉羊养殖基地。新建了雅士利应县公司、中粮应县糖厂、六味斋右玉公司、右玉中大科技等一批龙头企业，农业产业化水平进一步提高。扎实推进新农村建设。300个试点村和重点推进村全部完成了"四化四改"和"五个一工程"，18个试点村被评为省级示范村。2007和2008连续两年我市成为全省农村工作唯一项项获奖的市。

　　基础设施建设和生态文明建设力度空前，宜居城市建设迈出坚实步伐。中心城市建设大规模展开。围绕"北方生态园林城市"和最宜居最宜发展城市建设目标，累计投资近50亿元，新建改建了世纪大道、南环路。西环路、大运路市区段等十余务主干道，实现了城市外环成形、路街贯通，形成了158平方公里的城市道路大框架，城市建成区面积由16平方公里扩大到23平方公里。开工建设了图书馆、博物馆、体育馆、新闻大楼、艺术中心等一批重要公共建筑，城市功能进一步完善。大力实施了绿化、亮化、净化工程，城市建成区绿化覆盖率达到43.3始，基本形成了"一半森林一半城"的

　　格局，荣获"山西省园林城市"称号。特色城镇建设步伐加快。各县区都展开了大规模的旧城改造，完成投资30多亿元，县城面貌发生了翻天覆地的变化。公路建设持续推进。累计投资100多亿元，新建改建公路3900公里，每万人拥有公路里程全省第一;完成村通水泥路5209公里，行政村通达率达到87.2%。资源环境保护成效明显。完成了所有陶瓷企业的煤气烧成改造，建成了5家污水处理厂和市区垃圾处理厂，燃煤电厂全部安装了脱硫除尘设施，恢河、七里河市区段得到综合治理。万元地区生产总值综合能耗逐年下降，2008年节能降耗工作名列全省第一。二氧化硫、化学需氧量排放量逐年减少，市区空气质量二级以上天数2008年达到318天。造林绿化力度不断加大。累计造林198万亩，平均每年近40万亩，国家林业局把我市作为联系点，并向全国推广我市经验。广大人民群众切身感受到，我们的城市越来越漂亮，越来越宜居，一个山青水秀、绿树成荫的新朔州已经展现在我们面前!

　　各项社会事业蓬勃发展，事关群众切身利益的问题得到有效解决。坚持以人为本，抓住财力增长较快的有利时机，积极加快各项社会事业发展，并创造性地推出一系列改善民生的措施，使广大群众得到了更多实惠。五年来，市财政在教育、卫生、就业、社会保障四大民生领域累计投入61.3亿元。教育事业加速发展。"普九"任务圆满完成，"两免一补"政策全面落实。新改扩建农村寄宿制学校225所，并全面启动了寄宿制学校营养餐工程，全省寄宿制学校建设现场会在我市召开。教育质量明显提高，高考达线率逐年上升。高等教育迈出新步伐，职业技术学院建成使用，师范专科学校

　　开工建设。医疗卫生事业取得较大突破。公共卫生服务体系渐趋完善，市中心医院、急救中心、疾控中心和传染病医院相继建成，县乡村三级医疗卫生机构达标率达到73.6%，并在全省率先实现了城乡居民基本医疗保险全覆盖，群众看病难、看病贵问题得到有效缓解。就业形势保持稳定。新增就业岗位11.46万个，安排再就业人员3.98万名，帮助1.53万名困难人员实现就业。社会保障体系更加完善。社保基金由2003年的2.5亿元提高到12.5亿元。国家出台的十二项救助保障制度全部建立。在全国首家推出65岁以上农村居民生活补贴制度，12万农村老人享受到了公共财政的阳光。一个人人享有社会保障的局面正在形成。科技、文化、体育事业全面进步。组建了六个专家服务组，实施市级以上科技项目436项，获得省以上科技进步奖16项，建成国家级"可持续发展试验区"一个，粉煤灰综合利用研究成果全国领先。朔州秧歌、晋北道情、赛戏、梨花春酿造工艺成功申报国家非物质文化遗产保护项目。广场文化节和网络文化节连续举办。城乡公共体育设施大幅度增加，群众性体育活动广泛开展。市区有线电视数字化改造全面完成。人口和计划生育工作连续五年受到省政府表彰。精神文明建设成果丰硕，群众文化生活繁荣活跃。社会治安综合治理不断加强，有效维护了社会稳定。积极支持国防后备力量建设，双拥工作深入开展。审计监督作用得到充分发挥。统计、气象、档案史志、民族宗教、妇女、儿童、老龄、残疾人等各项社会事业都取得了新成绩。妥善处置突发事件，政府驾驭经济社会发展的能力得到加强。高度重视安全生产，扎实推进了煤炭行业"十关闭"、"十整顿"和打击非法开采专项行动，认真开展安全隐患大排查活动，建立健全了安全生产八项制度，安全生产形势逐年好转，2007和2008连续两年受到省政府表彰。加强应急管理体系建设，妥善处置了一系列突发性事件。圆满完成了抗击南方雨雪冰冻灾害保电煤、迎峰度夏保奥运和支援地震灾区抗震救灾任务，受到国家有关部门和省政府的充分肯定，为全省争了光。三鹿奶粉事件发生后，市政府投入1亿多元，在全国率先实行了奶牛补贴政策，开展了"放心奶进万家"活动，有效保护了奶农利益和奶业发展，得到国务院检查组的高度评价。

　　坚持依法行政，民主法制建设稳步推进。市政府自觉接受市人民代表大会及其常委会的监督，主动接受市政协的民主监督，认真听取各民主党派、工商联、无党派人士、人民团体的意见，办理人大代表建议意见175件，政协委员提案788件，做到了件件有回音，事事有落实。积极推行政务公开，加快政府职能转变，深入开展廉政建设。2007年我市被评为全国政务公开先进单位。各位代表，过去的五年，是我们在科学发展观指引下，进一步解放思想、锐意进取的五年;是经济大发展、社会大进步、人民生活显著改善的五年。五年来，我们经受住了一次次考验，渡过了一道道难关，实现了一个又一个重大跨越。五年间，我市多项主要经济指标增幅一直全省领先，多项重要工作走在全省前列。2007和2008连续两年在全省经济社会发展考核评价中被评为优秀市。去年全省十一个现场会在我市召开。同时，还铸就升华了朔州精神和右玉精神，成为激励我们永远前进的精神动力。过去五年的成就确实令人振奋。现在，我郑重向各位代表报告，我们不仅超额完成了市四届人大一次会议确定的各项目标，也提前三年实现了"十一五"规划确定的地区生产总值(现价)和财政总收入翻番目标，在实现"两个再翻番"和建设塞外最宜居最宜发展城市的伟大征程中迈出了坚实的一步!

　　各位代表，五年的成就来之不易。这是省委、省政府和市委正确领导的结果，是市人大、市政协及各民主党派、人民团体鼎力支持的结果，是全市人民团结进取、辛勤劳动的结呆。在此，我代表市人民政府，向为朔州改革开放和现代化建设作出重大贡献的全市人民，向所有关心、支持和参与朔州建设与发展的同志们、朋友们， 表示崇高的敬意和衷心的感谢!同时，我们也清醒地认识到，目前还存在着不少问题。受国际金融危机的影响，经济增长的速度明显放缓;支柱产业单一的局面没有得到明显改变，发展方式仍然比较粗放，节约资源和保护环境的任务仍很艰巨;安全生产形势依然严峻，安全工作压力仍然很大;农业基础仍很薄弱，农民持续增收难度加大;社会事业欠帐较多，一些涉及群众切身利益的问题还亟待认真解决;一些政府部门和工作人员还存在一定的官僚主义、形式主义。对这些问题，我们必须高度重视，认真加以解决。

　　二、对下届政府工作的建议

　　各位代表，未来的五年，是我们继续保持经济社会又好又快发展势头、加速推进现代化建设的关键时期，是圆满完成"十一五"规划、顺利实施"十二五"规划，承前启后、继往开来的重要时期，也是挑战与机遇、困难与希望相互交织的时期。我们将面对前所未有的金融危机给经济社会发展带来的严重影响，需要我们付出比以往任何时候都更大的努力。但同时也面临着前所未有的重大政策机遇和投资高峰，是我们进一步夯基础、强后劲、增实力的千载难逢之机。危机终将是短暂的，后劲和实力是永存的。只要我们坚定信心，敢于迎接挑战，善于抓住机遇，未来的五年，仍必将是充满胜利的五年，是全市经济社会更好更快发展的五年!

　　根据目前的形势和我市实际，建议下届政府工作的总体思路是:认真贯彻落实党的十七大精神，以邓小平理论、"三个代表"重要思想和科学发展观为指导，紧紧围绕转型发展、安全发展、和谐发展、全面发展的主题，坚持以农业现代化、工业新型化、城市园林化、政府高效化、人民生活小康化为方向，突出基础设施建设、产业优化升级、资源环境保护、改善民计民生、促进社会和谐五个重点，全面推进经济建设、政治建设、文化建设、社会建设和生态文明建设，着力提高全市综合经济实力、可持续发展能力、社会和谐水平和人民生活水平，为把我市建设成为塞外最宜居最宜发展城市而奋斗!

　　按照这一总体思路，今后五年，全市经济社会发展要力争实现"五个提升":

　　一是提升综合经济实力，建设繁荣富强新朔州。努力保持全市经济又好义快发展的大好势头，在圆满实现"十一五"后三年地区生产总值和财政总收入"两个再翻番"的基础上，再上一个新台阶。到2013年，全市地区生产总值力争突破1000亿元，比2008年增长1.4倍。财政总收入突破200亿元，比2008年翻一番。固定资产投资累计达到2000亿元，达到上一个五年的3.3倍。

　　二是提升转型发展质量，建设充满活力新朔州。生态畜牧和新型能源工业"两大基地"建设取得重大突破。农业农村经济实现半农半牧目标，奶牛饲养量达到20万头，比2008年增加5.6万头;鲜奶产量达到65万吨，增加22万吨。煤电工业技术水平显著提升，真正实现传统产业新型化。全市煤炭产量达到2亿吨，比2008年增长70%;电力装机容量突破1000万千瓦，比2008年增长1.2倍。食品、陶瓷、现代煤化工、新型材料、生物医药等新兴产业更加壮大，形成主导产业多元化的格局。服务业实现大发展，到2013年，服务业增加值达到330亿元，比2008年增长1.5倍。

　　三是提升可持续发展能力，建设环境优良新朔州。基础设施建设取得更大进展，全面完成有关铁路、公路、机场、引黄工程、大型水库建设，形成铁路、公路、航空立体交通网，根本解决水资源缺乏问题。城市功能进一步完善，承载能力进一步提高，城镇化水平达到50%，比2008年提高5个百分点。生态环境明显改善，大气污染、水污染得到有效控制。节能减排取得明显成效，万元地区生产总值综合能耗降到全国平均水平。植树造林投资每年达到10亿元以上，每年造林绿化33万亩以上，林木绿化率每年提高2个以上百分点，到2013年，全市林木绿化率达到31%，中心城市绿化覆盖率达到48%，建成国家级园林城市和省级宜居城市。

　　四是提升社会文明程度，建设和谐稳定新朔州。基础教育全面加强，建成1——2所在全省有较大影响的名牌中学，高中阶段毛入学率由80%提高到87%。高等教育取得更大发展，朔州师专和能源学院全部建成使用。职业教育体系基本完善。科技对经济增长的贡献率由43.7%提高到47%。各项社会事业更加繁荣，高标准完成一批文化、卫生、体育、广播电视设施建设，形成覆盖全市的市、县、乡三级网络。精神文明建设扎实推进，进入全国文明城市行列。政府行为规范高效，依法行政水平大为提高。民主法制建设进一步加强，社会治安状况进一步好转。

　　五是提升人民生活水平，建设富裕安康新朔州。城乡居民收入不断增加。2013年，城镇居民人均可支配收入提高到2.5万元月到2008年的1.8倍;农民人均纯收入提高到7300元，达到2008年的1.5倍;农村基本消除贫困，整体进入宽裕型小康。就业服务体系进一步健全，就业率明显提高。社会保障体系更加完善，保障水平大幅度提高，真正实现老有所养、学有所教、病有所医、住有所居、劳有所得。

　　做好今后五年的工作，要把握好以下五项原则:一是必须坚持科学发展。形势越严峻，推进科学发展的要求越迫切。要始终以坚持科学发展观为指导，做到以人为木，统筹兼顾，加快推进转型发展、安全发展、和谐发展、全面发展。二是必须坚持与时俱进。困难时期，既有掉队落伍、拉大差距的风险，也有重塑优势、加快掘起的机遇。我们必须顺应大势、把握机遇，认真研究国内外形势对我市经济发展的影响，研究有关政策对经济发展的推动作用，做到顺应形势变化、顺应发展实际、顺应群众期盼。三是必须坚持项目带动。充分利用国家增加投资、扩大内需的机遇，以项目建设为主线，集中实施一批重大基础设施项目、民生项目和"三农"项目，全力支持重大产业项目建设，强化经济增长的支撑力。四是必须坚持特色突破。紧紧围绕我市的资源优势、区位优势和产业优势，加大招商引资力度，推进特色产业发展，加快形成特色工业、特色农业、特色服务业等产业群、产业链，以特色促发展，以特色求突破。五是必须坚持改革创新。坚持把改革创新作为保增长、促发展的根本动力，在经济社会发展的各个方面，进一步解放思想，大胆探索，形成更具活力的体制机制。

　　各位代表，目标鼓舞人心，任务极其艰巨。我相信，只要全市上下瞄准目标不动摇，抢抓机遇不放松，团结拼搏，不懈努力，我们的宏伟蓝图就一定会变成美好现实!

　　三、2009年工作总体部署

　　今年是建国六十周年和我市建市二十周年，也是本届政府的开局之年，做好今年的工作，意义重大。

　　今年的核心任务是保增长、保民生、保稳定。全市经济社会发展的主要预期目标是:地区生产总值达到480亿元，增长8%;工业增加值完成270亿元，增长12%;全社会固定资产投资完成330亿元，增长35%;财政总收入达到111亿元，增长8.8%;一般预算收入达到41.2亿元，增长9%;社会消费品零售总额达到125亿元，增长20%;粮食产量稳定在12亿斤左右;城镇居民人均可支配收入达到15260元，增长9%;农民人均纯收入达到5160元，增长9%。

　　在完成好上述指导性指标的同时，确保完成以下约束性指标:万元地区生产总值综合能耗下降5.6%;二氧化硫、化学需氧量排放量分别下降到14.5万吨和1.15万吨;居民消费价格涨幅控制在4%以内;城镇登记失业率控制在4%以内;人口自然增长率控制在6%以内。

　　要重点抓好六个方面的工作:

　　(一)全力准迸重点工程建设，更加有力地拉动经济增长

　　要把搞好重点工程建设作为保持经济平稳较快发展的主抓手，以基础设施建设为重点，努力加大各个领域的投资力度，最大限度地发挥投资对经济增长的拉动作用。

　　全市年内实施和启动的重点工程项目有162个，总投资1304亿元。主要包括:铁路公路投资200亿元，农业水利投资46亿元，保障性住房投资32.3亿元，教育卫生投资19亿元，节能减排和生态建设投资24亿元，电力投资181亿元，现代化矿井投资374亿元。主要项目有:四条铁路，即准朔铁路朔州段、北同蒲韩家岭—应县—原平铁路朔州段、大同—运城—西安铁路朔州段、北同蒲大新—韩家岭扩能改造应急工程。三条高速公路，即大同至呼市高速公路右玉段、灵丘至平鲁高速公路朔州段、环城高速公路西南段。七条干线公路，即元元公路至鹅毛口运煤通道、广武至阳方口运煤通道、连接平川四县区的经济开发路、大忻线拓宽改造、虎山线拓宽改造、市区至右玉扶贫公路、应县至繁峙出口公路。一大水利工程，即引黄北干线。一大生态治理工程，即以桑干河上游为重点的首都水资源生态环境综合治理工程。两所大学，即朔州师专和能源学院。还有七大煤电项目。目前已有123个项目开工，完成投资81亿元。这些重点工程，既关系当前的经济发展速度，又关系未来的发展后劲，每一项都是功在当代、利在千秋的德政工程、惠民工程、经济腾飞工程。这样大规模的集中投资，前所未有。我们一定要紧紧抓住这个千载难逢的机遇，以百折不挠的精神、坚韧不拔的毅力、不获全胜誓不罢休的顽强意志，组织动员全市各级各部门，万众一心，攻坚决战，力争所有项目年内全部开工，明年底绝大部分建成，努力实现我市工业化、城市化、现代化水平的新飞跃!

　　(二)大力推进产业结构调整，加快发展方式转变

　　经济危机之时，正是结构调整之机。我们当前所面临的困难，虽有国际金融危机与国内经济周期性波动相互叠加的影响，但最根本的还是自身结构性、素质性等深层次矛盾的集中反映。我们必须着眼长远、着眼可持续发展，坚定不移地推进产业结构调整，坚定不移地推进发展方式转变，坚定不移地推进转型发展。

　　全面实施工业产业调整和振兴规划。按照国务院和省政府的总体部署，结合我市实际，尽快制定实施煤炭、电力、陶瓷、新型材料、食品、冶金化工、煤机装备制造等七大重点产业调整和振兴规划，全面推进工业强市战略。大力提升煤炭工业素质。一要全力打好兼并重组攻坚战，大幅度提高煤炭产业规模化、集约化水平。按照"总量适度、优化布局、改善结构、提升水平"和"关小上大、产能置换、有序建设"的原则，以单井产量不低于90万吨、企业规模不低于300万吨的标准，务必在年底之前将现有135座地方煤矿压减到65座。这是煤炭工业发展史上的一次革命，是提高煤炭工业素质、转变煤炭工业发展方式、提高可持续发展能力的一项根本性举措，是具有前瞻性、战略性的重大决策，是实现转型发展、安全发展、和谐发展、全面发展的关键所在。一定要作为一项重要的政治和经济任务，坚决予以完成。二要加快推进机械化升级改造，大幅度提高煤炭产业现代化、安全化水平。兼并重组后的所有矿井都要坚决取消炮采，明年年底前全部实行机械化综合开采，建成木质安全型矿井。三要加快大型洗选设施建设，提高煤炭市场竞争力。年内投资32亿元，新建葫芦堂、芦家窑等8座洗选能力300万吨以上的洗煤厂，扩大洗选能力，提高煤炭质量。四要加快一批大型矿井建设，扩大产业总体规模。积极支持平朔东露天煤矿等大型矿井建设，全面完成现有矿井改造任务，扩大煤炭生产能力。加快电力工业发展。抓好已开工的右玉2X30万千瓦煤砰石电厂、山阴金海洋2X30万千瓦煤秆石电厂和开发区2X30万千瓦热电联供电厂建设，力争神电平鲁2X60万千瓦电厂年内开工，做好同煤怀仁2X20万千瓦电厂的前期工作，尽快将我市建设成为在全国更具影响的煤电一体化新型能源工业基地。培育壮大新兴产业。陶瓷业要着力提高档次，增加品种。冶金化工业要按照节能环保的要求，加大技术改造力度。新型材料业要围绕提高资源利用率，积极发展以煤砰石、粉煤灰、工业废渣为原料的新型干法水泥和新型墙体材料，抓好精细高岭土和耐磨材料加工。食品工业要立足特色农畜产品，加大对小杂粮、肉制品等农畜产品的加工转化力度，特别要继续扶持乳品加工业。煤机装备制造业要服务主导产业，加快形成立足木地、辐射周边的产业规模。狠抓工业园区建设。按照有利于产业集聚和循环经济发展的要求，加快发展工业园区，将其打造成为招商引资和产业结构调整的主要平台。新建加工制造业项目原则上都要规划到园区，在城镇居民生活区范围内的各类工厂要逐步迁建到园区。要重点抓好平朔、神头电厂、经济技术开发区、金海洋、富甲工业园区、金城东工业园区、云东经济开发区建设，加快红旗牧场煤电新区和粉煤灰综合利用工业园区建设。千方百计帮助企业渡过难关。采取促进产销衔接、减轻税费负担、推动银企合作、提供贷款贴息、鼓励技术创新等多种措施，切实帮助企业解决实际问题，推动经济平稳较快增长。

　　强力推动服务业发展。按照全市总体规划，积极提升传统服务业，大力发展现代服务业，突出抓好旅游业、现代物流业和现代机电维修业，不断提高服务业在国民经济构成中的比重，尽快建成高增值、强带动、广就业"的新型服务业体系。加快山阴广武、应县木搭和龙首山、城区神头海、怀仁金沙滩、右玉杀虎口、平鲁北固山等旅游景区的保护开发。推动豪德贸易广场、开发区汽配和矿山设备物流中心、怀仁陶瓷物流中心等尽快投入运营，丈持中煤综采设备维修中心、小松矿山设备维修公司等项目加快建设。认真落实支持服务业发展的各项政策措施。降低服务业准入门槛，放宽对服务业企业的限制;对发展较好的项目要给予扶持奖励;对鼓励类服务业实行水价、气价、电价与工业同价;在土地、信贷等要素供给上，向鼓励类服务业倾斜。

　　振兴七大产业，加快发展服务业，是我市今后一个时期经济工作的主要任务。我们一定要变压力为动力，化挑战为机遇，不畏难，不松劲，坚持不懈地抓下去，务力构建一个多元产业齐头并进、全面振兴的新局面!

　　(三)切实加强"三农"工作，全面提升农业农村发展水平

　　始终把"三农"工作作为重中之重，切实加大对"三农"的投入力度，发展现代农业，增加农民收入，促进农村和谐，全面提高农业农村发展水平。

　　深化农村改革，加大强农惠农力度。按照依法、自愿、有偿的原则，引导农民合理有序地进行土地承包经营权流转，加大对种粮大户的扶持，发展规模经营。积极稳妥地推进集体林权制度改革。进一步增加对"三农"的投入，健全农业补贴制度，在执行好国家和省各项强农惠农政策的基础上，继续对小杂粮、甜菜、大棚种植、养殖小区建设及农业技术推广等进行奖励补贴。进一步完善"以煤补农、以矿带村、一企一村"的帮扶机制，探索建立以煤补农专项基金。大力发展特色现代农业，巩固雁门关生态畜牧经济区建设排头兵地位。要把积极应对严重旱情作为当前农村工作的当务之急，千方百计扩大灌溉面积，力争全年有个好收成。要把稳定奶牛养殖业作为加强生态畜牧基地建设的核心工作。奶牛业是历经三十年艰苦创业发展起来的特色产业，在经历风险之后，更要倍加呵护。要制定实施奶业振兴规划，推进奶牛规模化标准化养殖，增强产业素质和抗风险能力，推动奶业持续壮大。继续抓好人畜分离小区、良种推广、饲草开发、防疫检疫四大工程，今年省市县要共同投入6000多万元，支持农民新建50个奶牛养殖小区，实现40%以上奶牛入区饲养，并配套建设1300个青贮窖，改造完善28个兽医中心站。同时，市县财政贴息一年，支持农村信用联社按每头产奶牛2000元的额度向全市奶农提供一亿元贷款。鼓励农民成立奶牛合作社，以养殖小区为载体，实行统一良种繁育、统一防疫检疫、统一饲料供应、统一机械挤奶，确保鲜奶质量。继续开展"新品放心奶进万家"活动，帮助乳品企业渡过难关。要靠强有力的措施，保护好奶业这个主导产业。保护好生态畜牧基地这块响亮的牌子，保护好农民增收致富的这条路子!

　　加强农田水利基本建设，夯实农业发展基础。以中低产田改造为主，加强耕地综合生产能力建设，重点抓好山阴西盐池10万亩盐碱地综合开发。抓好水利基础设施建设四项工程。一是新水源工程，建设好海子湾、东石湖、耿庄、大梁、尚希庄五座水库。二是新建和续建桑干河、恢河等四处灌区节水工程，总面积4.5万亩。三是水保治理工程，治理面积60万亩。四是全面完成已开工的神头泉保护工程。

　　提高农业现代化水平，转变农业发展方式。推进农业机械化，重点抓好玉米机收、土豆机收、设施蔬菜和甜菜机收四项农机新技术的示范推广，提高农业生产效率。积极支持龙头企业发展，加大对古城集团、中粮应县糖厂、雅士利应县公司、六味斋右玉公司、汇源右玉公司等龙头企业的扶持力度，鼓励发展"公司十基地十农户"产业经营模式，提升农业产业化水平。建立新型社会化服务体系，加快合作经济组织建设，鼓励农民成立各类专业合作社、专业服务公司，发展壮大农民经纪人队伍，为现代农业发展提供有力支撑。以"五个全覆盖"为重点，进一步推进新农村建设。要力争在两年内实现具备条件的建制村通水泥(油)路全覆盖，中小学校舍安全改造全覆盖，县乡村三级卫生服务体系特别是村级卫生室全覆盖，村通广播电视全覆盖，农村安全饮水全覆盖，使广大农民共享改革发展成果。今年，在上级资金支持的基础上，市财政投入1.4亿元，全力推进这项工作。村通水泥路方面，建设1226公里，通达80个村，通达率达到92%。中小学校舍改造方面，平鲁、山阴、怀仁、应县、右玉今年要全部完成改造任务。农村卫生服务体系建设方面，新建12所中心乡镇卫生院和550个村卫生室，使村卫生室覆盖率达到92%。村通广播电视方面，平川村镇加快有线电视数字信号通达，边远山区年内全面完成卫星信号入户覆盖。农村安全饮水方面，要解决13万人的饮水困难。怀仁、应县力争年内率先实现"五个全覆盖"。继续加快新农村建设。新增新农村试点村120个，试点村总数达到420个，占到全市农村的四分之一。加快以沼气为主的可再生能源建设。按照新的标准，加大扶贫开发力度，继续抓好移民搬迁、整村推进、第二轮"两区"项目建设、劳动力转移和培训教育五项工程，培训农民10万人，转移就业3万人，移民搬迁3000人。

　　(四)以创建国家园林城市为目标，加快最宜居最宜发展城市建设

　　继续集中力量、加大投入，扎实推进城市基础设施和生态环境建设，完善城市功能，强化城市管理，提升城市形象，努力打造绿色、清洁、宜居、文明的新朔州。

　　强化中心城市建设。继续保持中心城市大规模建设的力度。对去年已开工的二十项重点工程，要保竣工，出精品，向建市二十周年献礼。今年要着力抓好四大工程。一是加快推进朔州古城改造。年内要完成30万平方米的民居、商铺建设，到2010年基本完成古城改造任务，使朔州古城成为集商贸、文化、休闲为一体的特色旅游城，实现老城与新区的平衡发展。二是大力推进"城中村"改造。加快小村、北邢家河、贺家河、西街等"城中村"的拆迁改造，实现城市的协调发展。三是继续推进造路建设。拓宽民福西街、开发北路，延伸开发南路、建设南路、古北西街、北新街等道路，实现城市"交通畅通无阻、四通八达。四是继续推进绿化、净化、亮化、美化工作。绿化工作重点抓好张辽路、民福东街立交桥两侧、安泰街、顺义路、灰谊街、振武街六条街道和七里河湿地公园、恢河公园、金沙公园、西山生态公园四大公园绿化，新增绿地面积70.6万平方米，绿化覆盖率提高1.7个百分点，达到45%。净化工作要狠下决心整治脏乱差问题，实行全天清洁，做到垃圾日产日清。亮化工作要整体推进，全面提高。美化工作重点突出红顶白墙的特色，完成开发路两侧房屋屋顶平改坡工程。同时，要切实加强城市管理。创新城市管理模式，积极推进市政建设管理和公用事业管理体制改革，加快老旧小区改造，强化社区管理、物业管理和集贸市场管理。大力提高市民文明意识，扎实推进文明城市建设，提升城市文化品味。各县区要继续加快旧城改造步伐，打造特色城镇。

　　狠抓环境治理。深入推进"蓝天碧水"工程，年内所有企业要全部完成减排达标任务，达标无望的一律关停。围绕桑干河上游生态环境综合治理，推进飞翔水泥厂、碳素厂、嘉利淀粉厂、朔州造纸厂、汇诚铁含金厂、汇诚热电厂等污染严重企业的搬迁关停。加快取缔市区自备小锅炉，进一步提高城市集中供热率和燃气普及率。建成右玉污水处理厂，开工建设平鲁、山阴、应县、怀仁、右玉5座生活垃圾处理厂，推进生活垃圾焚烧发电项目。市区空气质量二级以上天数要保持在300天以上。

　　加大植树造林力度。坚持生态优先，每个县区要规划建设两个以上万亩大片造林工程，全面完善大运高速公路、109国道等12条共1000公里通道绿化，继续搞好京津风沙源治理、退耕还林、"三北"防护林四期和天然林保护四项国家重点林业工程。全市造林面积达到33万亩以上。

　　各位代表，建市二十年，一座生机勃勃、欣欣向荣的园林城市己经掘起在塞外大地上，这是我们最大的成就、最大的骄傲。我们一定要爱城如家、精心建设，努力使朔州的天更蓝、水更清、山更绿、空气更清新，使我们的家园更美丽!

　　(五)加快各项社会事业发展，进一步改善民生民计

　　天下之大，民生为要。越是在困难的时候，越要体察民情、关注民生，真正做到发展为了人民，发展依靠人民，发展成果由人民共享。

　　坚持教育优先发展。新改扩建60所农村高标准寄宿制小学，25所农村寄宿制初中，使农村学生全部进入寄宿制学校就读。进一步扩大农村寄宿制学校营养餐覆盖面，提高生活补助标准。加快发展职业教育。在六县区分别新改扩建一所功能齐全的职业中学或职教中心。加快朔州师专建设，争取秋季投入使用;力争能源学院早日开工建设。

　　大力发展医疗卫生事业。加快推进县乡村三级医疗卫生机构达标进程，两年内实现"村覆盖、乡达标、县提高"目标。逐步改扩建各县区县级医院。加快城市社区卫生服务中心建设。落实计划生育奖励政策，稳定低生育水平。

　　千方百计促进就业。就业是民生之木。面对严峻的经济形势，要实施更加积极的就业再就业政策。大力实施创业就业工程，在市场准入、财税金融、经营用地等方面提供便利和优惠，鼓励更多劳动者成为创业者。加强就业服务体系建设，建立全市统一规范的人力资源市场，搭建新的就业服务平台。积极开辟多种渠道增加就业，发挥好政府投资和重大项目带动就业的作用。鼓励和引导企业履行社会责任，稳定就业岗位，努力做到不裁员、不减薪。

　　不断完善社会保障体系。进一步加大财政保障力度，扩大社会保障覆盖范围，提高统筹和保障水平。社保基金要增加支出1亿元，着力推进"四个提高"，即提高城镇居民医保覆盖率和待遇，提高企业退休人员基本养老金水平，提高新型农村合作医疗参合率和财政补助标准，提高城乡居民最低生活保障标准，让更多的群众得到更多的实惠。

　　大力推进保障性住房建设。全市要投资50亿元，新建经济适用住房58.9万平方米，9438套;廉租住房31.8万平方米，6362套;完成城镇居民棚户区改造231万平方米，25484套。继续实施农村危房改造，解决800户农村困难群众住房问题。

　　全面加强安全稳定工作。坚持安全优先，严格落实企业安全生产主体责任和政府安全监管主体责任，全面开展生产安全、交通安全、防火安全、校舍安全、水库安全、食品药品安全等所有重点行业和领域的安全专项整治，提高安全生产管理水平，提高企业职工安全生产意识和生产技能，夯实安全基础，提高安全保障能力。认真做好信访工作，切实维护群众合法权益。加强防灾减灾工作，进一步健全突发事件应急管理体制，有效应对各类突发事件。加强国防后备力量建设，扎实开展"双拥"工作。要以做好建国六十周年安保工作为核心，深入开展"平安朔州"创建活动，加强社会治安综合治理，健全社会治安防控体系，促进社会治安形势进一步好转，增强人民群众的安全感。

　　加强社会主义精神文明建设。要以庆祝建市二十周年为契机，积极开展群众性文化体育活动，大力弘扬地方优秀传统文化，大力宣传建市以来所取得的巨大成就，丰富群众精神文化生活，激励全市人民以更加奋发有为、更加昂扬向上的精神状态，展现新朔州新风貌!

　　(六)加强政府自身建设，创优发展环境

　　新一届政府受任于国际金融危机持续蔓延的特殊时期，责任重大，使命艰巨。必须深入贯彻落实科学发展观，紧紧围绕保增长、保民生、保稳定这个大局，加强政府自身建设。

　　积极转变政府职能，努力建设服务型政府。按照"政企分开、政资分开、政事分开"的要求，深化行政管理体制改革，理顺部门管理职能，努力形成权贵一致、分工合理、程序正当、高效便民的行政管理体制。按照公开透明的办事原则和建设阳光政府的要求，进一步推进政务公开。深化行政审批制度改革，进一步减少行政许可，减少审批项目，减少审批环节，缩短审批时限，提高行政效率。通过正确履行政府职能和全面高效的服务，创造良好的发展环境，促进经济增长、社会发展。

　　切实转变工作作风，努力建设勤政务实政府。我们正处于加快发展、攻坚克难的关键时期，前进中的难题需要破解，发展中的机遇需要抢抓。我们一定要更加振奋精神，以过硬的工作作风，保证各项决策真正落到实处，保证各项任务圆满完成。要大兴调查研究之风。坚持一切从实际出发，深入基层，深入一线，发现新问题，研究新情况，形成新思路，推出新举措，打开新局面。要大兴服务群众之风。坚持密切联系群众，时刻把群众的安危冷暖挂在心上，关心群众疾苦，倾听群众呼声，积极主动为群众排忧解难，最大可能地让群众得实惠。要以群众需求为第一信号，以群众满意为第一标准，当好人民公仆，建设人民满意政府。要大兴真抓实干之风。各级领导干部要深入实际，靠前指挥，雷厉风行干工作，身先士卒做表率。要建立健全抓落实的工作责任制，责任到人，落实到位。重要工作要倒排工期，倒逼进度，确保按期完成。要加大督促检查力度，确保各项工作抓紧抓实抓出成效。

　　坚持依法行政，努力建设民主法治政府。各级政府要自觉接受人大监督和政协民主监督，高度重视人民群众监督和新闻舆论监督。要实行科学民主决策，凡是重大决策都要深入调查研究，广泛征求意见。要坚持依法行政，依法办事，严格按照法定程序行使相力、履行职责。要不断加强政府法制建设，督促各级各部门全面落实行政执法责任制，规范行政执法行为。

　　狠抓反腐倡廉，努力建设清正廉洁政府。各级政府要认真落实党风廉政建设责任制，严格执行中央关于廉政建设的各项规定础持"标本兼治、综合治理、惩防并举、注重预防"的方针，扎实推进惩治和预防腐败体系建设。要坚持勤俭办事，坚决反对铺张浪费。在经济困难时期，更要厉行节约，减少一切不必要的开支。要继续深入开展煤焦领域反腐败斗争，坚决打击官商勾结，打击权钱交易严厉查处违法违纪案件。今年政府投资力度大，新上项目多，要加强对各级财政扩大内需和改善民生资金的管理，抓好重大工程、重点行业和重要环节的监管，做到财政资金运用到哪里，审计就跟主到哪里，监督就落实到哪里。

　　各位代表，经过二十年的发展，我们己经站到了一个新的历史高度，一个更加美好的未来等待着我们去描绘，一个更加富强的朔州等待着我们去创造。我们面临的任务极其艰巨而繁重，我们肩负的使命极其重大而光荣。让我们在中共朔州市委的坚强领导下，开拓创新，迎难而上，抢抓机遇，奋力赶超，全力推进转型发展、安全发展、和谐发展、全面发展，在建设塞外最宜居最宜发展城市的伟大征程中迈出更加坚实的步伐，谱写更加辉煌的篇章!
